# Intimate Partner Violence against Mastectomized Women: Victims’ Experiences

**DOI:** 10.3390/curroncol29110674

**Published:** 2022-11-10

**Authors:** Franciéle Marabotti Costa Leite, Andreia Gomes Oliveira, Bruna Lígia Ferreira de Almeida Barbosa, Mariana Zoboli Ambrosim, Neiva Augusta Viegas Vasconcellos, Paulete Maria Ambrósio Maciel, Maria Helena Costa Amorim, Lorena Barros Furieri, Luís Carlos Lopes-Júnior

**Affiliations:** 1 Graduate Program in Public Health, Health Sciences Center, Federal University of Espírito Santo (UFES), Vitoria 29075-910, ES, Brazil; 2 Prefeitura Municipal de Vitória, Vitoria 29050-945, ES, Brazil; 3 Western New South Wales Primary Health Network-WNSWPHN, Dubbo, NSW 2830, Australia; 4 Nursing Department, Federal University of Espírito Santo (UFES), Vitoria 29075-910, ES, Brazil

**Keywords:** breast neoplasms, violence against women, mastectomy, violence

## Abstract

Exposure to situations of domestic violence during the treatment for breast cancer may compromise the treatment and quality of life of women patients, so it is essential that health professionals act in tracking this phenomenon in the approach to and care of women with breast cancer. The purpose of this study was to examine experiences of violence against women by their intimate partners after mastectomy. This is an exploratory descriptive study, with a qualitative approach, carried out in the Rehabilitation Program for Mastectomized Women in a Brazilian reference hospital for oncological treatment. Semi-structured interviews were conducted with 16 mastectomized women. For data analysis, a content analysis technique was performed. The women interviewed were predominantly brown, with a minimum age of 44 years and maximum of 72 years. They presented with low education, were married, and had a mean period of five years of breast cancer diagnosis. The participants reported that after mastectomy, they experienced episodes of violence at a time when they were extremely vulnerable due to the various cancer treatments. Three major thematic categories emerged from interview data across the data collection: (1) experiences of psychological violence, (2) experiences of physical violence, and (3) experiences of sexual violence. Psychological violence took the form of humiliation and contempt for their condition. Physical violence involved assault and sexual violence in the form of forced sex by coercion. Violence was a phenomenon present after mastectomy, practiced in the domestic environment by the intimate partner. We emphasize the importance of health professionals in screening for this issue by listening to and welcoming women, recording cases, exposing this situation, and contributing to prevention.

## 1. Introduction

A global public health problem, cancer places a high psychosocial and economic burden on individuals, families, and health systems [1]. The projections of the World Health Organization for the period 2018 to 2040 are 29.5 million new cases for all types of cancer across all ages and both sexes [2]. Among the chronic noncommunicable diseases, malignant neoplasms are the second leading cause of death in developed countries and are among the top three causes of death in adults in developing countries [3,4].

The latest report on the global burden of cancer, using the GLOBOCAN 2020 estimates of cancer incidence and mortality produced by the International Agency for Research on Cancer, which focuses on geographical variability in 185 countries worldwide, anticipated an incidence of 19.3 million new cases of cancer and 10 million deaths for 2020 [4]. The report pointed out that the most frequently diagnosed cancer and the main cause of death from cancer vary substantially between countries and within each country, depending on the degree of economic development, social factors, and lifestyle [4]. Breast cancer is the most common cancer among women globally. In 2018, there were 2.1 million new cases, equivalent to 11.6% of all estimated cancers. This value corresponds to an estimated risk of 55.2/100,000 [4,5]. 

According to data from the latest estimate made by the National Cancer Institute José Alencar Gomes da Silva (INCA), 625,000 new cases of cancer are expected to occur in Brazil for each year of the triennium 2020–2022 [6]. Specifically for women, 66,280 new cases of breast cancer are anticipated for each year of that triennium, which corresponds to an estimated risk of 61.61 new cases per 100,000 women. Excluding non-melanoma skin tumors, female breast cancer is the most common in all Brazilian regions, with an estimated risk of 81.06/100,000 in the Southeast region [6]. In the state of Espírito Santo, for the triennium 2020–2022, 790 cases of female breast cancer per 100,000 inhabitants are forecast [6]. 

The treatment of breast cancer has made substantial advances in recent years, resulting in the increase in the overall and relative survival rate of patients with this neoplasm. A good prognosis for breast cancer is directly related to early diagnosis, the rapid initiation of treatment, and technological advances in therapy, such as measures for early detection; personalized care; multidisciplinary, interdisciplinary, and specialized teams; combined protocols; target-molecular therapy; and the progress of clinical and translational research in oncology [7,8,9,10,11,12,13]. Currently, the 5-year relative survival rate for breast cancer can vary from 72% to 100% depending on staging, early detection, and type of treatment received in a timely manner and in specialized centers [6,7,10]. 

The diagnosis and treatment of breast cancer damage women’s daily lives, especially in relation to their sexuality, femininity, and body image [13]. In that sense, the psychological suffering women go through transcends the suffering of the disease itself, since it is linked to representations and meanings attributed to the disease throughout the history and culture and enters dimensions of the feminine being, interfering many times in the woman’s interpersonal relationships [14]. 

In this context, the family is very important, and the revelation of a diagnosis of cancer, although not always unexpected, is a difficult experience that causes feelings of deep sadness. Each family member reacts in a different way, with feelings of shock, fear, anguish, sadness, or even insecurity due to the stigma attributed to cancer as a painful and incurable disease [15].

It is therefore important to highlight that a healthy family relationship can help provide women with a favorable environment in which to face breast cancer, since any demonstration of care and attention coming from the children and the partner are only beneficial [16]. Reinforcing this statement, a recent study on the perceptions of breast cancer and its repercussions on daily life shows that breast cancer leads to significant changes in a couple’s lives and that mutual support is essential for better coping with the pathology, followed by family support [17].

It is important to highlight that women generally receive the diagnosis of breast cancer without their partners. This scenario is maintained throughout the treatment, perpetuating a condition in which the husband is sidelined in all the phases, from the diagnosis to the end of the treatment. This situation hinders the emotional support for the woman, since the partner collaborates in the process of psychological adaptation to the breast cancer [18].

The participation of the partner in all the stages is fundamental, since it will lead to an understanding of the process, enabling the partner to contribute to the reduction in the negative repercussions of breast cancer in the sexual, psychological, and social spheres [18,19]. 

A recent systematic review on exposure to violence among breast cancer patients showed how much this phenomenon causes harm to the victim [19]. Women diagnosed with breast cancer are victims of violence, have a higher occurrence of depression, as well as have damage to their physical, emotional, and functional well-being, which contributes to a worse prognosis of the neoplasm. In addition, it is important to highlight the underreporting of violence in the group of women with breast cancer, as this topic is still a taboo among patients, making it even more difficult to reveal it [19].

Exposure to situations of domestic violence during the treatment for breast cancer may compromise the treatment and quality of life of women patients, so it is essential that health professionals act in tracking this phenomenon in the approach to and care of women with breast cancer [19]. 

Hence, this study aimed to examine women’s experience of violence against them by their intimate partner after mastectomy.

## 2. Materials and Methods

### 2.1. Ethical Approval

The research project was approved by the Research Ethics Committee (CEP) of the Federal University of Espírito Santo under number 2,207,822. All ethical criteria were met, respecting the recommendations of Resolution 466/2012, which refers to research involving human beings.

### 2.2. Study Design

This was a descriptive study with a qualitative approach, conducted in a Rehabilitation Program for Mastectomized Women (PREMMA), which operates in a Brazilian reference hospital for oncological treatment in the municipality of Vitória, Espírito Santo state, in the Southeast Region of Brazil. 

### 2.3. Participants and Recruitment

The participants were 16 women diagnosed with breast cancer who had submitted to mastectomy, following the criterion of data saturation, which occurs when no new element is found, and the addition of new information is no longer necessary because it does not change the understanding of the phenomenon studied. This is a criterion that allows the validity of a data set to be established in qualitative studies [20].

### 2.4. Data Collection

The women were invited to participate in the research after receiving care from the nursing sector offered by the Program. It is important to highlight that only those who signed the Informed Consent Form were admitted into the study, after the purpose of the study had been explained to them and they had been advised of their freedom to withdraw at any time. Only the researcher and the interviewee participated in data collection. 

The interviewers were female, health professionals, who were not part of PREMMA and who have extensive experience in studies with a qualitative approach.

The interviews were carried out with the application of semi-structured interviews that required sociodemographic data and the following guiding question: “After breast cancer, did you experience violence from your intimate partner?” A pilot study was conducted with ten women in order to verify the suitability of the instruments for conducting the research. The data from this pilot study were not included in this research.

At the end of the interview, each participant received a folder explaining the phenomenon of violence against women and the networks of protection.

### 2.5. Data Analysis

The characterization data (age, education, marital status, family income, and time of diagnosis) of the participants were recorded and analyzed by obtaining measures of raw and relative frequency. The data concerning the women’s reports were recorded, transcribed, and analyzed according to the content analysis technique proposed by Bardin [21]. This analysis includes a set of systematic procedures to describe the content of messages in order to enable inference of knowledge related to the conditions of production/reception of these messages, covering the steps of pre-analysis, exploration of the material, treatment of results, and interpretation [21]. The narratives of the women interviewed were categorized into three thematic units on the basis of their experience of violence: (1) experiences of psychological violence, (2) experiences of physical violence, and (3) experiences of sexual violence. In order to preserve the anonymity of the women interviewed, the code I was used for “interviewee” followed by a number; thus, I1 was used to refer to interviewee number 1. 

## 3. Results

Sixteen mastectomized women participated in the study. The minimum age was 44 years and the maximum 72. Most had an incomplete elementary school education, a partner, and a family income of 1 to 2 minimum wages; the mean time of diagnosis was five years (Table 1). 

The interviewed women’s statements were grouped into three thematic categories depending on their experience of violence: (1) experiences of psychological violence, (2) experiences of physical violence, and (3) experiences of sexual violence.

The analysis of the data related to the guiding question: “After the breast cancer, did you start to experience situations of violence on the part of your intimate partner?” The interviewees’ narratives revealed that 50.0% experienced psychological violence, 30% experienced physical violence, and 20.0% experienced sexual violence.

### 3.1. Experiences of Psychological Violence

With regard to the experience of psychological violence, the comment of I1, married and diagnosed a year ago with breast cancer, indicate the presence of this problem practiced by the intimate partner, who sees the treatment as unnecessary, relating cancer to death.

*[...] Sometimes inside the house he would say: there, you are taking treatment for nothing, you are really going to die [...] (I1).*

For I12, diagnosed 10 years ago, the diagnosis of breast cancer and the surgery generated changes in the relationship with her partner, as she reported:

*[...] From the moment he (the husband) found out I had cancer, that I had breast surgery, he changed completely. He kept saying things to humiliate me, like, “Oh, you are not my wife, I have no wife like that, thin, bald, without both breasts” (pause for crying) [...] (I12).*

Breast cancer is a stigmatizing disease, which places female body image, especially after a mastectomy, in opposition to the parameters imposed by society, of what is expected of the female body. I3 reported having been deprecated as “mutilated.” A statement such as this reveals the degree of psychological violence by the intimate partner as a result of the breast removal surgery.

*[...] I was totally despised when I was “mutilated,” right. Mutilated in the breasts... The first time I took off my blouse near him, he said that if he had known that “they were going to” cut me like that, he would have done it himself. (pause)... sometimes I was changing my clothes and he called me a “cripple” [...] (I3).*

Participant I8 used resources based on coping and focused on emotion to deal with psychological violence; that is, she “pretended” not to be experiencing such a situation.

*[...] What struck me most in all this was the contempt. The worst thing he did was that. I pretended not to hear, but it hurt. It hurts. Sometimes, if it was a stranger, it wouldn’t hurt me so much [...] (I8).*

### 3.2. Experiences of Physical Violence

In this category, there were reports of physical aggression by the intimate partner, with incidents that ranged from a pinch, a push, or a punch to the use of a knife as a weapon. Despite the physical vulnerability due to the treatment, there was confrontation with and mastery over fear of the situation in pursuit of the preservation of their physical integrity, with the intervention of neighbors, as shown in the reports:

*[...] He pinched me and pushed me. I faced him and said that I am not afraid, I am not afraid of dying, I am not afraid of anything [...] (I1).*

*[...] There was a moment when he pulled the wig off my head and burned the wig [...] (I8).*

*[...] He came out of his room with a knife, when I went to get up, he came to punch me I got up and he came with the knife [...] (I12).*

The interviewees expressed indignation when questioning the justice of the application of the Maria da Penha Law, given the payment of bail for the release of the aggressor.

*[...] I didn’t have the physical strength to fight with him. Then it got to the point where he beat me and the neighbors “got involved” and he “went” to jail, but when he got there, he paid bail and got out because to justice, a life is nothing, it’s nothing [...] (I12)*

### 3.3. Experiences of Sexual Violence 

Reports of sexual practices without consent, which characterizes sexual violence, were present. It was reported by women who, because of fear or economic dependence, felt coerced by their intimate partner to submit to a sexual act.

*[...] He came to “get me” and go up against me by force. He said either I gave in or he would not buy anything else for the house [...] (I1).*

*[...] I slept with my room locked, but three times he broke the door down and forced me to have sex. I did it for fear of him doing something worse than what he was already doing to me, understand? [...] (I12)*

*[...] I had sex out of fear [...] (I6).*

## 4. Discussion

This study aimed to uncover the violence against women practiced by their intimate partners after mastectomy. The analysis of the statements revealed three thematic categories: (1) experiences of psychological violence, (2) experiences of physical violence, and (3) experiences of sexual violence.

Intimate partner violence (IPV) is a public health concern. A study conducted with users of primary care in the municipality of Vitória, Espírito Santo, Brazil, revealed the prevalence of psychological, physical, and sexual violence perpetrated against women by their intimate partners in the last 12 months: 25.3% (95% CI 22.6–28.2), 9.9% (95% CI 8.1–11.9), and 5.7% (95% CI 4.3–7.3), respectively [22]. The data indicate that this is a topical problem in Brazil, not only because of its magnitude, given the significant number of women affected, but also because of the social problems generated by gender violence, which implies the weakened autonomy of women affected by a relationship of domination and control by their partner [23].

A study conducted with women with breast cancer revealed that psychological violence was the most prevalent, with the partner cited as the main aggressor and the house the most frequent place in which the violence was perpetrated [24]. As noted in the present research, expressions of humiliation and feelings of fear and low self-esteem, as well as contempt exhibited by the intimate partners, reinforce how much psychological violence is present in the daily lives of women who have undergone mastectomies, with the partner as the most commonly cited aggressor.

It is important to emphasize that the experience of violence involves a range of feelings, often ambiguous and contradictory. The victims live between fear, anger, indignation, and surprise in relation to the aggressive actions of their partners, but the violence is perceived as negative [25]. Even so, the naturalization of violence, especially within the domestic space, is legitimized by male domination. This violence, marked by power over and oppression of women, leads us to reflect on the definitions and typology of violence against women emphasized by the Maria da Penha Law in Brazil; this reflection could facilitate a (re)conceptualization of violence in the unequal power relations that circumscribe the cruel dynamics in affective and marital relationships [26].

The breakup of a violent relationship can take years, given that many women continue with their partners due to financial dependence, fear of dying, waiting for a change in their partner’s behavior, the shame of assuming the failure of the relationship, or emotional dependence [27]. In the absence of economic factors, aspects such as intimacy and the centrality of the relationship can function to prevent the termination of the relationship [28]. Many women fail to report violence because they have the perception that they are not entitled to autonomy over their lives, because they believe they are guilty of the violence suffered, or because they do not even realize they are in a violent relationship [29].

A study conducted with 553 women diagnosed with breast, cervical, or colorectal cancer showed that domestic violence negatively influenced all health indicators related to cancer, suggesting that the identification of IPV and other stressors can provide important information to health professionals in order to contribute to the better planning of assistance, disruption of violence, and improvement in the well-being of these women [30].

The present study noted the experience of sexual violence in the reports of participants who highlighted coerced sexual practices, committed without consent, or motivated by fear of their partner. These results show how fundamental it is that health professionals take into consideration the complex interaction between the cultural, relational, and subjective aspects of the sexual experience after breast cancer in order to provide better care in the context of oncological assistance [31,32,33,34,35,36,37,38,39,40].

Provision of comprehensive care to women with breast cancer experiencing violence requires the construction of a network of services to confront that violence, this network being one of the most important and challenging strategies for dealing with a problem that is complex and multifaceted, so that the network contributes to the strengthening of victims and professionals, and so that they will feel supported and encouraged to act [41].

It is worth considering as a limitation of this study the fact that it did not investigate whether the women had already experienced tensions that interfered with their relationships prior to the disease. However, this does not prevent us from concluding that there is a need for professionals to assist these women and to provide holistic care capable of uncovering previous or current cases of violence, which are often omitted by women because they feel inhibited, ashamed, or too insecure to report what has happened, in order to contribute to their comprehensive care and record their cases, playing an important role in their care and the prevention of this phenomenon.

## 5. Conclusions

This is one of the few studies that we know of that has approached violence against woman in a context of the great vulnerability that is the experience of mastectomy resulting from a diagnosis of breast cancer. It was observed that the physical, sexual, and psychological violence practiced by their intimate partners may be present in this phase, considered a time of great need for family and social support.

The results of this study reaffirm the importance of health professionals in the care of women with breast cancer, and especially those in situations of violence. Health professionals have a role of immense relevance not only in the reception of victims, but also and especially in the recording of this problem, giving women the opportunity of inclusion in a network of protection and care and thereby enabling the removal of this phenomenon. It is important to emphasize that it is essential that women be assisted by a multidisciplinary and interprofessional team, given the complexity of violence and the demands that arise in different bio-psycho-social areas resulting from the experience of this serious public health phenomenon that is violence against women.

## Figures and Tables

**Table 1:** Participants’ characteristics. Vitória, Espírito Santo, Brazil, 2018.

Codification	Age	Education *	Marital Status	Family Income **	Time of Diagnosis (Years)
I1	52	2	Married	1	1
I2	64	3	Divorced	1	3
I3	62	1	Married	1	10
I4	68	4	Married	3	9
I5	47	1	Married	2	3
I6	47	1	Married	2	7
I7	45	1	Married	1	<1
I8	47	1	Stable union	1–2	1
I9	52	1	Married	3–4	4
I10	55	1	widow	2	4
I11	44	1	Married	3	<1
I12	56	3	Married	1–2	10
I13	72	3	Married	2	15
I14	49	1	Single	2	5
I15	46	3	Married	1	<1
I16	55	1	Married	1–2	6

I = Interviewee; * Illiterate = 1, Incomplete Elementary = 2, Complete Elementary = 3, Higher Education = 4; ** In Brazilian minimum wage. Brazilian minimum wage corresponds to USD 231.73 (quote on 15 September 2022).

## Data Availability

Data is available from the corresponding author upon reasonable request.

## References

[1] Siegel R.L., Miller K.D., Fuchs H.E., Jemal A. (2022). Cancer statistics, 2022. CA Cancer J. Clin..

[2] World Health Organization (2021). Cancer Management. Geneva: WHO. https://www.who.int/cancer/en/.

[3] Torre L.A., Siegel R.L., Ward E.M., Jemal A. (2016). Global Cancer Incidence and Mortality Rates and Trends—An Update. Cancer Epidemiol. Biomark. Prev..

[4] Sung H., Ferlay J., Siegel R.L., Laversanne M., Soerjomataram I., Jemal A., Bray F. (2021). Global Cancer Statistics 2020: GLOBOCAN Estimates of Incidence and Mortality Worldwide for 36 Cancers in 185 Countries. CA Cancer J. Clin..

[5] Ferlay J., Colombet M., Soerjomataram I., Mathers C., Parkin D.M., Piñeros M., Znaor A., Bray F. (2019). Estimating the global cancer incidence and mortality in 2018: GLOBOCAN sources and methods. Int. J. Cancer.

[6] Brasil (2019). Instituto Nacional de Câncer José Alencar Gomes da Silva. Coordenação de Prevenção e Vigilância. Estimativa 2020: Incidência de câncer no Brasil. https://www.inca.gov.br/sites/ufu.sti.inca.local/files/media/document/estimativa-2020-incidencia-de-cancer-no-brasil.pdf.

[7] Lopes-Júnior L.C., Lima R.A.G. (2019). Cancer care and interdisciplinary practice. Cad. Saude Publica.

[8] Lopes Júnior L.C. (2021). The era of precision medicine and its impact on nursing: Paradigm shifts?. Rev. Bras. Enferm..

[9] Lopes-Júnior L.C., Olson K., de Omena Bomfim E., Pereira-da-Silva G., Nascimento L.C., de Lima R.A. (2016). Translational research and symptom management in oncology nursing. Br. J. Nurs..

[10] Abrahão C.A., Bomfim E., Lopes-Júnior L.C., Pereira-da-Silva G. (2019). Complementary therapies as a strategy to reduce stress and stimulate immunity of women with breast cancer. J. Evid. Based Integr. Med..

[11] Lopes-Júnior L.C. (2021). Personalized Nursing Care in Precision-Medicine Era. SAGE Open Nurs..

[12] Amorim M.H.C., Lopes-Júnior L.C. (2021). Psychoneuroimmunology and nursing research: Discovery, paradigm shifts, and methodological innovations. Acta Paul. Enferm..

[13] Oliveira F.B.M., Santana e Silva F., Prazeres A.S.B. (2017). Impacto do câncer de mama e da mastectomia na sexualidade feminina. Rev. Enferm. UFPE on line.

[14] Silva L.C. (2008). Breast cancer and psychological suffering: Female-related aspects. Psicol. Estud..

[15] Oliveski C.C., Girardon-Perlini N.M.O., Cogo S.B., Cordeiro F.R., Martins F.C., Paz P.P. (2021). Experience of families facing cancer in palliative care. Text. Context. Enferm..

[16] Vale C.C.S.O., Dias I.C., Miranda K.M. (2017). Breast cancer: The impact of mastectomy on women’s psyche. Mental.

[17] Ferreira D.B., Farago P.M., Reis P.E.D., Funghetto S.S. (2011). Our life after breast cancer: Perceptions and repercussions from the perspective of the couple. Rev. Bras. Enferm.

[18] Finck C., Barradas S., Zenger M., Hinz A. (2018). Quality of life in breast cancer patients: Associations with optimism and social support. Int. J. Clin. Health Psychol..

[19] Aygin D., Bozdemir H. (2019). Exposure to violence in breast cancer patients: Systematic review. Breast Cancer.

[20] Nunes N.L.C., Vignuda S.T., Santos O.I.C., Moraes J.R.M.M., Aguiar R.C.B., Silva L.F. (2018). Saturação teórica em pesquisa qualitativa: Relato de experiência na entrevista com escolares. Rev. Bras. Enferm..

[21] Bardin L. (2016). Análise de Conteúdo.

[22] Leite F.M.C., Amorim M.H.C., Wehrmeister F.C., Gigante D.P. (2017). Violence against women, Espírito Santo, Brazil. Rev. de Saúde Pública.

[23] Chacham A.S., Jayme J.G. (2016). Violência de gênero, desigualdade social e sexualidade: As experiências de mulheres jovens em Belo Horizonte. Civ.-Rev. de Ciências Sociais.

[24] Luvisaro B.M.O., Gradim C.V.C. (2016). Violence against Women with Breast Neoplasms. J. Pharmacy Pharmacol..

[25] Moura M.A., Oliveira P.R.F. (2000). A percepção das mulheres vítimas de lesão corporal dolosa. Esc. Anna. Nery.

[26] Guimarães M.C., Pedroza R.L.S. (2015). Violência contra a mulher: Problematizando definições teóricas, filosóficas e jurídicas. Psicol. Soc..

[27] Brasil (2005). Secretaria Especial de Politicas para as Mulheres. Enfrentando a Violência contra a Mulher.

[28] Giordano P.C., Soto D., Manning W.D., Longmore M.A. (2010). The characteristics of romantic relationships associated with teen dating violence. Soc. Sci. Res..

[29] Pazo C.G., Aguiar A.C. (2012). Sentidos da violência conjugal: Análise do banco de dados de um serviço telefônico anônimo. Physis.

[30] Coker A.L., Follingstad D., Garcia L.S., Williams C.M., Crawford T.N., Bush H.M. (2012). Association of intimate partner violence and childhood sexual abuse with cancer-related well-being in women. J. Womens Health.

[31] Vieira E.M., Santos D.B., Santos M.A., Giami A. (2014). Vivência da sexualidade após o câncer de mama: Estudo qualitativo com mulheres em reabilitação. Rev. Latino-Am. Enferm..

[32] Vaziri S.H., Lotfi Kashani F. (2012). Sexuality after breast cancer: Need for guideline. Iran. J. Cancer Prev..

[33] Maleki M., Mardani A., Ghafourifard M., Vaismoradi M. (2022). Changes and challenges in sexual life experienced by the husbands of women with breast cancer: A qualitative study. BMC Womens Health.

[34] Carroll A.J., Baron S.R., Carroll R.A. (2016). Couple-based treatment for sexual problems following breast cancer: A review and synthesis of the literature. Support. Care Cancer.

[35] Santos D.B., Santos M.A., Vieira E.M. (2014). Sexuality and breast cancer: A systematic literature review. Saúde Soc..

[36] Brajkovic L., Sladic P., Kopilaš V. (2021). Sexual Quality of Life in Women with Breast Cancer. Health Psychol. Res..

[37] Ooi P.S., Draman N., Muhamad R., Yusoff S.S.M., Noor N.M., Haron J., Hadi I.S.A. (2021). Sexual Dysfunction Among Women With Breast Cancer in the Northeastern Part of West Malaysia. Sex. Med..

[38] Dinapoli L., Colloca G., Di Capua B., Valentini V. (2021). Psychological Aspects to Consider in Breast Cancer Diagnosis and Treatment. Curr. Oncol. Rep..

[39] Lopes-Júnior L.C. (2022). Cancer symptom clusters: From the lab bench to clinical practice. Rev. Bras. Enferm..

[40] Lopes-Júnior L.C., Tuma M.C., Amorim M.H.C. (2021). Psychoneuroimmunology and oncology nursing: A theoretical study. Rev. Esc. Enferm. USP.

[41] Hasse M., Vieira E.M. (2014). How health professional assist women experiencing violence? A triangulated data analysis. Saúde Debate.

